# Effectiveness of a long-term use of a minimalist footwear versus habitual shoe on pain, function and mechanical loads in knee osteoarthritis: a randomized controlled trial

**DOI:** 10.1186/1471-2474-13-121

**Published:** 2012-07-12

**Authors:** Francis Trombini-Souza, Ricardo Fuller, Alessandra Matias, Mariane Yokota, Marco Butugan, Claudia Goldenstein-Schainberg, Isabel C N Sacco

**Affiliations:** 1 Department Physical Therapy, Speech, and Occupational Therapy, School of Medicine, University of São Paulo, Cidade Universitária, Rua Cipotânea 51, 05360-160, São Paulo, São Paulo, Brazil; 2 Rheumatology Division, School of Medicine, University of São Paulo, São Paulo, Brazil

**Keywords:** Osteoarthritis, Knee, Footwear, Shoe, Foot biomechanics, Gait

## Abstract

**Background:**

Recent studies have shown an important reduction of joint overload during locomotion in elderly women with knee osteoarthritis (OA) after short-term use of minimalist shoes. Our aim is to investigate the chronic effect of inexpensive and minimalist footwear on the clinical and functional aspects of OA and gait biomechanics of elderly women with knee OA.

**Methods/Design:**

Fifty-six elderly women with knee OA grade 2 or 3 (Kellgren and Lawrence) are randomized into blocks and allocated to either the intervention group, which will use flexible, non-heeled shoes— Moleca®—for six months for at least six hours daily, or the control group, which could not use these shoes. Neither group is undergoing physical therapy treatment throughout the intervention period. Moleca® is a women’s double canvas, flexible, flat walking shoe without heels, with a 5-mm anti-slip rubber sole and a 3-mm internal wedge of ethylene vinyl acetate. Both groups will be followed for six months and will be assessed at baseline condition, after three months, and after six months (end of intervention). All the assessments will be performed by a physiotherapist that is blind to the group allocation. The primary outcome is the pain Western Ontario and McMaster Universities Osteoarthritis (WOMAC) score. The secondary outcomes are global WOMAC score; joint stiffness and disability WOMAC scores; knee pain with a visual analogue scale; walking distance in the six-minute walk test; Lequesne score; amount and frequency (number of days) of paracetamol (500 mg) intake over six months; knee adduction moment during gait; global medical assessment score; and global patient auto-assessment score. At baseline, all patients receive a diary to record the hours of daily use of the footwear intervention; every two weeks, the same physiotherapist makes phone calls to all patients in order to verify adherence to treatment. The statistical analysis will be based on intention-to-treat analysis, as well as general linear models of analysis of variance for repeated measure to detect treatment–time interactions (α = 5%).

**Discussion:**

This is the first randomized, clinical trial protocol to assess the chronic effect of minimalist footwear on the clinical and functional aspects and gait biomechanics of elderly women with knee osteoarthritis. We expect that the use of Moleca® shoes for six months will provide pain relief, reduction of the knee adduction moment when walking, and improve joint function in elderly women with knee OA, and that the treatment, thus, can be considered another inexpensive and easy-to-use option for conservative OA treatment.

**Trial registration:**

NCT01342458

## Background

Osteoarthritis (OA) is the most common musculoskeletal disease [1,2], mainly affecting joints that suffer constant overload, such as the knee. As a consequence, routine daily activities, such as walking and ascending and descending stairs, as well as individual autonomy, may be compromised.

Knee adduction moment (KAM), usually used to estimate intra-articular overload, has been strongly related to OA severity [3] and its progression [4,5]—the higher the intra-articular load, the more severe will be the progression and functional consequences of the disease.

Great efforts have been made to improve conservative OA treatments, as, according to the Osteoarthritis Research Society International (OARSI) [6], the actual available pharmacotherapy is insufficient to control the clinical manifestations of OA. Surgical procedures have a high potential to reduce symptoms and improve function, but they are associated with increased costs, prolonged work impairment, and complications. According to the OARSI, conservative treatments, such as physical measures that allow improvement of biomechanical aspects and minimize intra-articular load, particularly KAM, produce the best evidence of efficacy. Braces for knee misalignments [7,8] and foot splints [9-13] are commonly used and frequently recommended as important interventions for the management of knee OA.

More recently, it has been suggested that foot flexibility during barefoot gait has the power to reduce the KAM in individuals with OA [14,15]. However, the opportunity to perform daily activities barefoot is rare and may be uncomfortable, especially for elderly people. There is scientific evidence that modern shoes, including the high-heeled, rigid-soled shoes commonly used by the majority of women in their everyday lives [16], generate higher knee moments and instability during locomotion. This is because modern shoes provide less flexibility, thereby not reproducing the degrees of freedom of the foot when it is bare, and causing progressive damage to knee joints with OA [17].

It has been shown that minimalist shoes that are capable of simulating barefoot gait reduced KAM in patients with knee OA [17-19]. More recently, we have shown that the acute use of an inexpensive, flexible, non-heeled footwear reduced significantly KAM in elderly women with OA during gait [20] and descending stairs [21].

Although it is reasonable to suppose a therapeutic effect of flexible, minimalist, and inexpensive shoes in the treatment of knee OA, its efficacy in reducing pain and KAM, as well as recovering musculoskeletal function in elderly people with knee OA has not been tested yet through a randomized, controlled trial. If effective, the use of these inexpensive, minimalist shoes might be part of a treatment prescription, representing a way of reducing costs associated with OA patients’ treatment.

Our hypothesis is that the use of the intervention footwear by elderly women with knee OA for at least six hours per day for six months could (i) relieve knee pain; (ii) improve musculoskeletal function in daily living activities; (iii) reduce knee load during gait; and (iv) reduce the use of medication, and, therefore, could be considered another inexpensive option for conservative OA treatment. Our aim is to investigate the therapeutic effect of inexpensive and minimalist footwear on the clinical and functional aspects of OA and gait biomechanics in elderly women with knee OA.

## Methods/Design

### Overview of the research design

A randomized, controlled trial was designed to study the effects of the shoe intervention on knee OA. Elderly women diagnosed with knee OA are recruited from hospitals and primary care centres, and are referred to a physical therapist, who performs the group allocation. The participants then are referred to another physical therapist, who performs the initial blind assessment.

All patients allocated to the intervention group (IG) receive the minimalist footwear on the first day and have to use it for six months, for at least six hours daily. The patients allocated to the control group (CG) do not receive the intervention footwear. All patients are assessed at baseline condition, after three months, and after six months (end of intervention). During this period, they continue to receive routine analgesic medication recommended by the medical staff at the hospital.

### Participants and recruitment

This study is currently recruiting patients (study start date: March 2011).

The eligibility criteria are:

Women 60 to 80 years of age

OA diagnosed according the American College of Rheumatology - ACR [22] criteria

Radiographic evidence of medial femorotibial classified as Kellgren–Lawrence [23] grade 2 or 3

Absence of diagnosed hip and/or ankle OA, rheumatoid arthritis, or systemic inflammatory arthritis; asymptomatic OA of both knees

Knee pain between 3 and 8 on a VAS

Body mass index (BMI) <35 kg/m^2^[17]

No history of surgical procedure on knee, ankle, or hip, and no muscle injury in the last six months

Absence of diagnosed neurological disease [24]

Leg length discrepancy smaller than 1 cm

Absence of rigid hallux [12]

Absence of arthroplasty and/or lower limb orthoses or indication of lower limb arthroplasty throughout the intervention period

Currently not using the Moleca® or similar shoes for more than 25 hours per week

No knee intra-articular steroid or hyaluronic acid three and six months, respectively, before inclusion in the study [12]

Absence of knee instability (verified by clinical examination to assess the collateral and cruciate ligaments)

Absence of dementia or inability to give consistent information [24]

Ability to walk independently without an assistive device for at least six hours a day

No physical therapy or acupuncture treatment throughout the intervention period

The use of paracetamol 500 mg every four hours is permitted for both groups for the control of pain, according to ACR recommendations for OA treatment [25]. NSAIDs and DMARDs are allowed if initiated at least eight and four weeks, respectively, prior to randomization, and they must be used in stable doses throughout the study [12].

**Figure 1:**
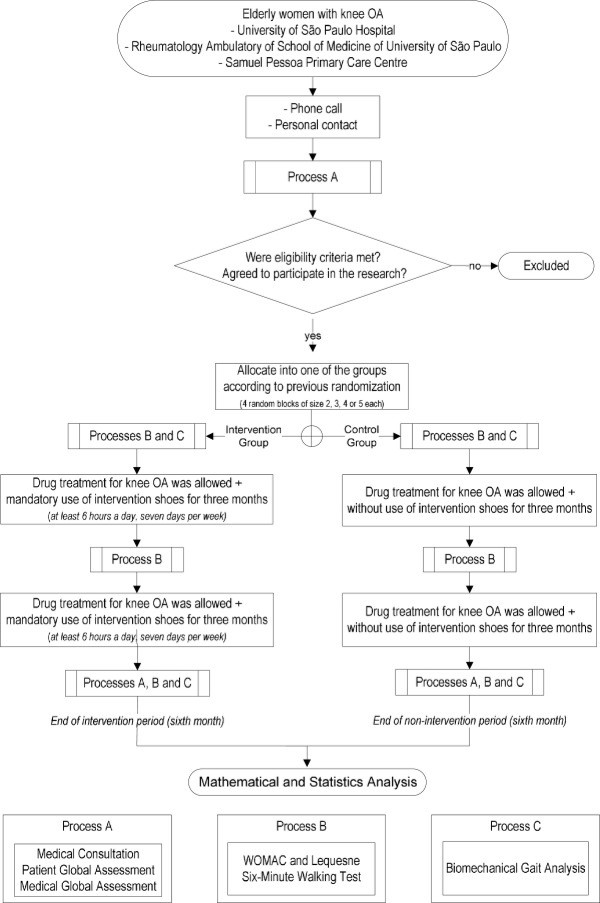
Flowchart of the protocol steps.

The participants are recruited from three settings: (a) rheumatology clinic medical care located at the Hospital das Clínicas of the School of Medicine at the University of São Paulo, (b) orthopaedic ambulatory medical care located in the University Hospital, and (c) a primary care centre at the School of Medicine of the University. All potential patients are interviewed by telephone and, when selected, are assessed, in the rheumatology clinic of the Hospital das Clínicas of the School of Medicine at the University of São Paulo, by a rheumatologist who is blind to the patient’s allocation (Process A). The design and flowchart of the steps of the protocol are presented in Figure 1.

After the recruitment, the patients are submitted to the inclusion and exclusion criteria by the physician (Process A). All patients are assessed in the biomechanics laboratory (Processes B and C) by a physiotherapist. This assessment represents the baseline condition. After three months, both groups are assessed according to Process B. At the end of the intervention period (after six months), both groups are assessed according to the complete protocol (Processes A, B, and C) (Figure 1).

### Randomization and blinding

The randomization schedule was prepared using Clinstat software [26] by an independent researcher who was not aware of the numeric code for the control and intervention groups. A numeric block randomization sequence is kept in opaque envelopes.

After the patients’ agreement and assignment to participate in the research, the allocation into the groups is made by another independent researcher, who is also unaware of the codes. Only the physiotherapist responsible for the clinical trial knows who is receiving the intervention.

The initial and final clinical and medical examinations (Process A) are carried out by a rheumatologist who is blind to the patient’s allocation. One physical therapist (PT 2), who is blind to the treatment allocation, is responsible for all clinical, functional, and biomechanical assessments (Processes B and C). Another physical therapist (PT 1) is responsible for group allocation and monitoring the use of the intervention footwear by telephone. Both physiotherapists are blind to the block size used in the randomization procedure. The trial statistician is blind to treatment allocation until the main treatment analysis has been completed. To ensure that the researcher physiotherapists remain blind, they will emphasize to patients that they should not be told whether or not the patient has received the intervention footwear.

### Protocol assessments

Eligible patients are assessed according to Process A, which consists of an interview and a clinical examination to classify the patient’s overall health condition, performed by the rheumatologist (global medical assessment score 1–5) and by the patient (global patient self-assessment score). Full-extension anterior–posterior knee radiographs are used to confirm the OA diagnosis and classification according to Kellgren–Lawrence criteria [23]. Process A is performed by a rheumatologist who is blind to the patient’s allocation. The two overall health condition assessments are conducted at baseline and at the end of the intervention period by the same physician.

At baseline, patients from both groups receive a diary to record the amount, in hours, of Moleca® use, and the intake of paracetamol tablets (500 mg) on a daily basis. Every three months, the diaries are collected in order to account for the Moleca® and painkillers use (Table 1).

**Table 1:** Diary of intervention shoe use and Paracetamol intake

**NAME:____________________________________________________________________MONTH ** – First day:_____/_____/_____Final day: _____/_____/_____															
**DATE**	/	/	/	/	/	/	/	/	/	/	/	/	/	/	/
**Hours of use**															
**Amount of tablets (paracetamol)**															
**DATE**	/	/	/	/	/	/	/	/	/	/	/	/	/	/	/
**Hours of use**															
**Amount of tablets (paracetamol)**															

**INSTRUCTION:** At the end of every day, you must write down the total amount of hours that you used the Moleca and the number of paracetamol tablets taken (take only if you feel knee pain).

Process B consists of an anamnesis specific to knee pain symptoms at night, while standing, and during locomotion, using a VAS. Musculoskeletal function is analyzed by a six-minute walk test [27], the Western Ontario and McMaster Universities Osteoarthritis questionnaire (WOMAC) [28] and the algofunctional Lequesne Index of severity of OA [29]. All patients are assessed according to Process B at baseline, after three months of intervention, and at the end of six months intervention, by PT 2. The WOMAC questionnaire assesses three dimensions: pain, disability, and joint stiffness in knee OA, and it is refering to 72 hours prior to the evaluation, using a 24-question, 5-point Likert protocol. The higher the score, the worse is the condition [28]. The Lequesne questionnaire assesses pain or discomfort, maximum distance walked, and activities of daily living, 72 hours prior to the evaluation [29]. The higher the score, the worse is the condition. The six-minute walk test [27] is a practical, simple test that measures the distance that a patient can quickly walk on a flat, hard, indoor surface in a period of six minutes. This test evaluates the patient's ability to perform daily living activities. We strictly followed the American Thoracic Society guidelines when conducting this test [27].

Process C consists of a biomechanical gait analysis; the inverse dynamic approach is employed to calculate KAM. Patients of both groups are assessed at the baseline and at the end of six months. We use three-dimensional displacement of passive reflective markers (20 mm in diameter), tracked with six infrared cameras (OptiTrack FLEX: V100; Natural Point, Corvallis, OR, USA) [20] and ground reaction forces acquired by a force plate (AMTI OR 6–7 1000, Watertown, MA, USA) embedded in the centre of a 10-meter walkway. The reflective markers are placed on the subject using a standard Cleveland Clinic marker set [30]. Extra markers are placed bilaterally at the medial knee joint line, medial malleolus, and first metatarsal joint for the static standing trial, in order to determine relative joint centres of rotation for the knee, ankle, and longitudinal axis of the foot. These extra markers are removed in the gait trial. In addition, two sets of technique clusters are placed in the lateral thigh and over the shank. Marker placement is performed by the same physiotherapist who performs the blind assessment (Processes B and C). We evaluate the limb correspondent to the symptomatic knee in subjects with unilateral OA and the most symptomatic knee in subjects with bilateral OA [17,18].

The laboratory coordinate system is established at one corner of the force plate, and all initial calculations are based on this coordinate system. Each lower limb segment (foot, shank, and thigh), based on surface markers, is modelled as a rigid body with a local coordinate system that coincides with the anatomical axes. Translations and rotations of each segment are reported relative to neutral positions defined during the initial standing static trial. The inertial properties are based on Dempster’s standard regression equations. The moment of inertia and location of centre of mass are computed assuming the thigh and shank segments as a cylindrical geometric shape. The knee is examined as a bicondylar type joint with biplane movements; flexion/extension, adduction/abduction (in smaller range of motion) andmedial and lateral axial rotation [31].

Force and kinematic data acquisition are synchronized and sampled by an A/D card (AMTI, DT 3002, 12 bits) at 100 Hz. We use the Visual3D software (C-motion, Kingston, ON, Canada) and a custom-written Matlab function (MathWorks, Natick, MA, USA) to perform all mathematical procedures.

### Outcome measures

The primary outcome measure is the pain WOMAC score (Table 2), based on the OARSI task force [32].

**Table 2:** Outcome measures

**Primary Outcome**	**Measurement**
Pain subscale	WOMAC osteoarthritis score
**Secondary Outcomes**	
- Stiffness and disability subscale	- WOMAC osteoarthritis score
- Knee pain	- Visual Analogue Scale (0–10 cm)
- Function and progression of OA	- Lequesne’s algofunctional questionnaire score
- Six-minute walk test result	- Distance (m)
- Paracetamol (500 mg) intake	- Days and amount of paracetamol ingested over six months
- Knee load during gait	- Knee adduction moment (%body weight/height) by inverse dynamics during gait
- Global medical assessment	- Ordinal scale (1-much worse, 2-slightly worse, 3-no change, 4-slightly better, 5-much better)
- Global patient’s self-assessment	- Ordinal scale (1-much worse, 2-slightly worse, 3-no change, 4-slightly better, 5-much better)

The secondary outcomes are: total WOMAC score; joint stiffness and disability WOMAC subscale scores; knee pain symptom on movement, at rest, and at night with a VAS; walking distance in the six-minute walk test; Lequesne score; intake of paracetamol (500 gm) (number of days and quantity) over six months; knee load during gait; global medical assessment score; and global patient self-assessment score (Table 2).

### Intervention

The intervention is based on the daily use of the intervention footwear—Moleca®—for six months, for at least 42 hours per week (approximately six hours a day, seven days a week).

**Figure 2:**
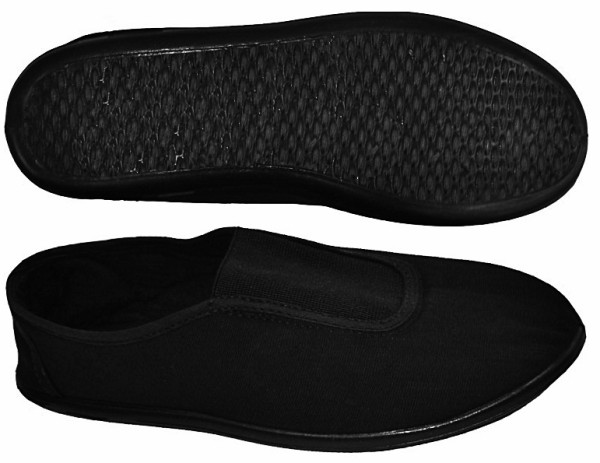
.** Moleca® shoes for women, made of double canvas, with flexible rubber soles and without heels.**

Moleca® footwear (Calçados Beira Rio S.A., Novo Hamburgo, RS, Brazil) is a low-cost women’s double canvas, flexible, flat, walking shoe without heels, with a 5-mm anti-slip rubber sole and a 3-mm internal wedge of ethylene vinyl acetate (Figure 2). Its mean weight is 0.172 ± 0.019 kg, ranging from 0.091 to 0.182 kg depending on the shoe size. This minimalist footwear has been produced on a large scale in Brazil since 1986 and is commonly worn by the elderly of lower social classes. We bought all the pairs of Moleca® shoes for the patients. For hygiene reasons, we ask the patients to wear thin cotton or silk socks with the footwear.

Every two weeks, the same physiotherapist (PT 1) makes phone calls to the IG patients in order to verify adherence to the treatment and the correct filling out of the diary. After three and six months of intervention, the patients in the IG group have their pair of Moleca® shoes photographed and checked for natural wear of the shoe with its daily use. If there are tears or holes in the shoes, they are replaced with a new pair.

During the intervention period, patients from the CG should not wear Moleca® or similar minimalist shoes, but they continue to receive their recommended health care and painkiller medication at the hospital. At the end of the intervention period, all CG patients also receive a free pair of Moleca® shoes.

### Sample size and statistical analysis

The sample size calculation was made based on the achievement of a minimal clinically meaningful improvement [33] using an effect size of 0.30 (moderate effect size), considering the pain WOMAC score as primary outcome measure. We took the SD estimates from a study we had previously completed, in which we recruited a similar patient cohort [18]. A sample size of 56 patients is needed to provide 80% power to detect a moderate effect difference between the highest and lowest group pain means, assuming an alpha level of 0.05 and a statistical design of F test of repeated measures (between and within effects), and assuming a 10% loss to follow-up. Twenty-eight patients will be allocated to each group. The statistical analysis will be based on intention-to-treat analysis, and general linear models of analysis of variance for repeated measure will be used to detect treatment–time interactions (α = 5%). The outcome measures will be compared among baseline, three months, and six months, and between groups. The expected treatment effect is a difference of 20% between groups in the percentage of patients improved, using the WOMAC score [34].

### Ethics and data security

This trial was approved by the Ethics Committee of the School of Medicine of the University of São Paulo (# 0810/10). All patients are asked to provide written, informed consent prior to randomization, using standard forms. Data access and storage are kept with National Health and Medical Research Council guidelines, as approved. This trial is registered in Clinical Trials (a service of U.S. National Institutes of Health) with the number NCT01342458.

## Discussion

Based on our previous and promising results of an efficient reduction of knee joint load in women both with and without OA during activities of daily living using the same footwear [20,21], we propose a conservative and low-cost intervention for these patients. It is important to emphasize that this type of footwear has been available in our country on a large scale since the 1980s, and it is widely used for the comfort it provides. Therefore, it is expected that adherence to this form of therapy will be greater and easier than the use of knee braces [7,8] and subtalar strapping splints [9].

To our knowledge, this is the first randomized, clinical trial that aims at treatment for knee OA by means of inexpensive and minimalist footwear. We propose a flexible shoe as an intervention that mimics barefoot gait and, consequently, may reduce knees load in elderly women with chronic OA. If effective, at the end of the intervention, we expect to observe knee pain relief, improvement of musculoskeletal function in daily living activities, and reduced intake of analgesics by elderly women with knee OA.

In contrast to previous studies that include different levels of radiographic OA involvement [35-37], we emphasize herein the importance of selecting a more homogeneous group of patients, based on the Kellgren–Lawrence radiographic scale (grades 2 and 3). In fact, heterogeneity among radiographic involvement within the intervention and control groups could be associated with a variety of levels of symptoms and therefore, different effectiveness of the proposed intervention. Furthermore, only patients with pain in the 3–8 range of the VAS are included, in order to eliminate participants who probably will not achieve further improvement, as well as participants with more severe degrees of the disease, who probably will be less responsive.

Another issue taken into account was the higher BMI limit. In our study, the maximum permitted BMI was 35 km/m^2^. Although the KAM was normalized by a percentage of the subject’s body weight and height to allow for comparisons among subjects, high concentrations of fat in some parts of the body, such as the thigh and the pelvis, can provoke many movement artefacts and then interfere in KAM calculation.

In this study, we highlight the crucial importance of periodic monitoring of the use of the intervention footwear and medication intake. One physical therapist has telephone contact with the participants every two weeks, to know about the use and the physical state of the intervention shoes, the filling out of the diary, and any issues related to the OA or medication intake. Usually, this practice is not observed in other studies [38,39] and may have interfered in their results. In these cited studies, the participants were asked only to record and return, via a packet sent in the mail, the number of hours they wore the study shoes per day, without monitoring regularly their usage.

Taking into account the period of daily use, we stipulated that the Moleca® shoe should be utilized for at least six hours daily, corresponding to 75% of the hours spent in usual daily activities. To determine this time usage, we took into account the analysis of van Raaij et al. [37], who determined the ideal timing of daily splint use, including insoles and braces, for knee OA. However, these same authors consider that the optimum amount of time for the use of the studied shoe cannot be categorically determined. Patients that have previously used Moleca® shoes for at least 25 hours weekly are excluded from this study in order to avoid bias in the results analysis.

The primary outcome measure is the pain WOMAC score, based on the OARSI task force [32]. This primary outcome, obtained by means of the WOMAC questionnaire, is widely used in the evaluation of knee OA. It is a valid, reliable, and responsive outcome measure that has been used as a main outcome in other randomized clinical trials in knee OA [39,40], and it is more sensitive to treatment changes than other WOMAC dimensions [28]. We used a linguistically validated Portuguese questionnaire [41].

Of our many secondary outcomes, we emphasize KAM. This variable is very important in representing knee loads during locomotion. In contrast to the other variables, which consist of subjective aspects, such as pain, stiffness, and disability, KAM is not subject to this drawback.

We expect that this trial will provide additional insights regarding the effectiveness of minimalist and inexpensive footwear intervention in the improvement of clinical and functional aspects of OA and gait biomechanics in elderly woman with knee OA. Moreover, it may be indicated as another inexpensive option for conservative treatment of elderly women with knee OA.

## Competing interests

The authors affirm that this study has not received any funding/assistance from a commercial organization that could lead to a conflict of interest, nor do they maintain any commercial or professional relationships with the manufacturers of the footwear utilized in this study.

## Authors’ contributions

All authors have made substantial contributions to all three of the following sections: The conception and design of the study, acquisition of data, or analysis and interpretation of data. Drafting the article or revising it critically for important intellectual content. Final approval of the version to be submitted. All authors read and approved the final manuscript.

## Pre-publication history

The pre-publication history for this paper can be accessed here:

http://www.biomedcentral.com/1471-2474/13/121/prepub
